# Exploring Astragaloside IV in Ischemic Heart Disease: A Comprehensive Systematic Review and Meta‐Analysis of Preclinical Cardiotoxicity Models

**DOI:** 10.1002/jbt.70365

**Published:** 2025-06-23

**Authors:** Alosh Greeny, Gollapalle Lakshminarayanashastry Viswanatha, Rekha Raghuveer Shenoy, Shylaja Hanumanthappa, Dinesh Kumar Chellappan, Jagnoor Singh Sandhu, Saumya Khanna, Nandakumar Krishnadas

**Affiliations:** ^1^ Department of Pharmacology, Manipal College of Pharmaceutical Sciences Manipal Academy of Higher Education (MAHE) Manipal Karnataka India; ^2^ Independent Researcher Bangalore Karnataka India; ^3^ Department of Life Sciences, School of Pharmacy IMU University Kuala Lumpur Malaysia; ^4^ Centre of Medical and Bio‐Allied Health Sciences Research Ajman University Ajman United Arab Emirates; ^5^ Central Animal Research Facility, Kasturba Medical College Manipal Academy of Higher Education Manipal Karnataka India; ^6^ Center for Animal Research, Ethics and Training (CARET) Manipal Academy of Higher Education Manipal Karnataka India

**Keywords:** antiapoptosis, anti‐inflammatory, Astragaloside IV, cardioprotection, ischemic heart disease

## Abstract

This systematic review and meta‐analysis were conducted to evaluate the therapeutic efficacy of Astragaloside IV (As‐IV) in ischemic heart disease based on the preclinical evidence and to correlate the cardioprotective effect with various available mechanisms. This systematic review and meta‐analysis were conducted based on the results of a thorough literature search in databases of published papers, such as PubMed, Embase, and Google Scholar. A total of 18 studies that met the inclusion/exclusion criteria were included. The meta‐analysis has shown the significant therapeutic efficacy of As‐IV on ischemic heart disease. As‐IV has decreased the myocardial infarction size, the left ventricular weight indices, the left ventricular internal diameter in systole, and the left ventricular internal diameter in diastole. As‐IV has decreased the level of the third type of collagen and the decreased activity of creatine kinase and lactate dehydrogenase. Also, As‐IV has markedly decreased the rate of apoptosis and the expression of the proapoptotic markers such as caspase‐3 and Bax. The left ventricular systolic pressure, as well as the arterial shortening edge and the ejection fraction, has increased. The levels of the antiapoptotic protein Bcl‐2 increased. In addition, As‐IV has a powerful anti‐inflammatory influence by inhibiting the main markers of inflammation, such as TLR4, IL‐1, TNF‐α, and TGF‐β. As‐IV has also caused an effect on angiogenesis by increasing the VEGF level. The results have revealed the As‐IV, as a decent universal medicine for ischemic heart disease because of its variety of actions and effectiveness.

## Graphical abstract

This systematic review and meta‐analysis evaluated the therapeutic efficacy of Astragaloside IV in ischemic heart disease, encompassing 18 preclinical studies. The findings demonstrated that As‐IV significantly reduced myocardial infarction size and apoptosis while improving cardiac function and angiogenesis through various mechanisms, along with potent anti‐oxidant, anti‐apoptotic, and anti‐inflammatory effects. These results suggest that As‐IV is a promising universal treatment for ischemic heart disease.

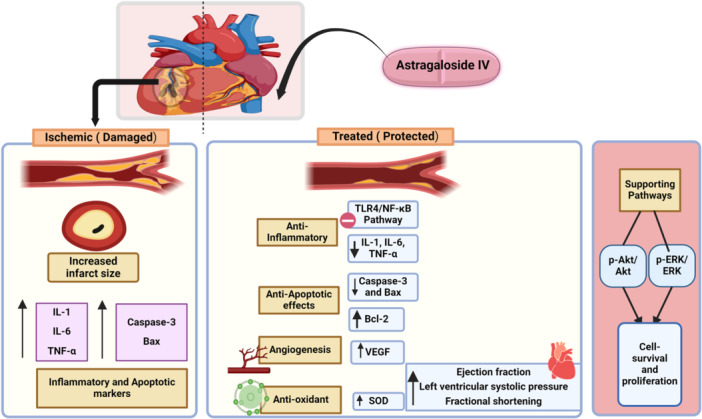

**Abbreviations**
As‐IV: Astragaloside IV
Akt: protein kinase B
ATP: adenosine triphosphate
Bax: Bcl‐2 associated X‐protein
Bcl‐2: B‐cell lymphoma
BW: body weight
CAD: coronary artery disease
CI: confidence interval
CK: creatinine kinase
EF: ejection fraction
ERK: extracellular signal‐regulated kinase
FS: fractional shortening
IHD: ischemic heart disease
IL: interleukin
IV: inverse variance
LDH: lactate dehydrogenase
LVEDP: left ventricular end‐diastolic pressure
LVIDs: left ventricular internal dimension—
  systole
LVIDd: left ventricular internal dimension—diastole
LVSP: left ventricular systolic pressure
LVW: left ventricular weight
MA: meta‐analysis
MI/R: myocardial ischemia–reperfusion
MeSH: Medical Subject Headings
NF‐ κB: nuclear factor κB
PI3k: phosphatidyl inositol‐3‐kinase
PTEN: phosphatase and tensin homolog, deleted on chromosome 10
RCT: randomized controlled trial
RevMan: Review Manager
SOD: superoxide dismutase
SR: systematic review
TCM: traditional Chinese medicine
TGF‐β: transforming growth factor –β
TLR: toll‐like receptors
TNF‐α: tumor necrosis factor‐α
VEGF: vascular endothelial growth factor

## 1 Introduction

Ischemic heart disease (IHD) is a severe pathological condition wherein the blood flow to the myocardium is decreased, causing a supply–demand mismatch in myocardial oxygen. The leading cause of IHD in most cases is CAD, where the epicardial coronary arteries are blocked due to the buildup of atherosclerotic plaque. Hence, IHD and CAD are interchangeable [1]. IHD is a progressive disorder. However, it can turn unstable if the plaque ruptures, leading to acute coronary syndrome [1, 2]. In western countries like the United States, CAD accounts for 30% of all deaths above the age of 35 [3], and in Europe, it accounts for about 20% of all deaths annually [4].

As mentioned, the leading cause of IHD is CAD. The atherosclerotic plaques are driven by risk factors such as smoking, inflammation, male gender, diabetes mellitus, history of atherosclerotic disease, hypertension, renal impairment, and ancestral health background of premature IHD [5, 6]. These risk factors accelerate the plaque buildup in the coronary arteries, thus narrowing the lumina and reducing the blood flow. This causes endothelial dysfunction, resulting in a supply–demand mismatch of myocardial oxygen and ischemia [1]. Among all the risk factors mentioned above, smoking and hypertension are the main contributors to the rise in IHD cases. Elevated cholesterol levels have a direct correlation with IHD.

The occurrence of IHD is far greater in developed countries than in developing and underdeveloped nations. The developed regions include northern parts of America, Asia Pacific, western Europe, Australia, and the Middle East. The prevalence rates are at the highest among countries like Latvia and Estonia [7]. However, with respect to the total number of cases, countries with the highest number of inhabitants, like India and China, have the highest burden. India has more than 37 million cases in total. Another 4.7 million cases are expected to be added to this number each year [6]. This is most likely due to unhealthy lifestyle and dietary patterns. The primary endpoint of IHD is heart failure.

The most popular drugs for the treatment of IHD are β‐blockers, nitrates, and antiplatelets. The β‐blockers block the β1‐receptors in the heart and reduce the heart rate, contractility of the myocardium, and blood pressure, while the nitrates act by releasing nitric oxide (NO), which activates guanylate cyclase in vascular smooth muscle, leading to increased cyclic GMP and subsequent vasodilation, thereby reducing preload and myocardial oxygen demand. Further, the antiplatelet agents, such as aspirin, inhibit platelet aggregation by irreversibly blocking the cyclooxygenase‐1 (COX‐1) enzyme, reducing thromboxane A2 synthesis and preventing thrombus formation in coronary arteries.

As‐IV is the active component of *Astragali radix*, a prevalent traditional Chinese medicine (TCM). According to the 2020 edition of the Chinese Pharmacopoeia, *Astragalus* refers to the dried root of the legume species *Astragalus mongholicus* or *Astragalus capsularis* [8]. Astragaloside IV (As‐IV) is a tetracyclic triterpene saponin with a lanosterol‐like structure, derived from *Astragalus membranaceus* (*A. membranaceus*). While As‐IV exhibits limited solubility in water, it is soluble in organic solvents such as methanol, ethanol, and acetone. The molecular formula of As‐IV is C_41_H_68_O_14_, with a molecular weight of 784.97 Da, and various studies have reported beneficial pharmacological effects of As‐IV on various disease conditions [9]. Ample number of studies are available exploring the beneficial effects of As‐IV on different diseases, including the nervous system, cardiovascular system, kidney disease, gynecological diseases, lung disease, and diabetes. As‐IV has been shown to inhibit the activation of microglia, as reported by Yao et al. [10], thereby preventing the release of pro‐inflammatory cytokines. This action further inhibits neutrophil and NK cell infiltration and NLRP3 inflammasome activation, demonstrating neuroprotective effects through its anti‐inflammatory activity [10]. Additionally, As‐IV has exhibited beneficial effects in diabetic kidney disease through antioxidant and anti‐inflammatory mechanisms, reduction of endoplasmic reticulum stress, and regulation of calcium homeostasis [11]. According to Wen et al. [12], As‐IV enhances autophagy by activating the PPAR‐γ pathway, which influences the proliferation and apoptosis of ovarian granulosa KGN cells, leading to improved ovarian function in rats with polycystic ovary syndrome (PCOS) [12].

Furthermore, As‐IV has demonstrated protective effects on lung function and anti‐pulmonary fibrosis activity by inhibiting the GTP–GDP domain of RAS, thus downregulating the RAS/RAF/FoxO signaling pathway. This study suggests that As‐IV is a promising natural candidate for protecting against pulmonary fibrosis in chronic obstructive pulmonary disease (COPD) [13]. In skeletal muscle cells (C2C12 myotubes), As‐IV enhances glucose transport by activating the insulin receptor substrate (IRS)1/protein kinase B (AKT) pathway, while inhibiting the IκB kinase (IKK)/inhibitor‐κBα (IκBα) pathway [14]. Additionally, Lv et al. demonstrated that As‐IV could lower blood glucose levels in diabetic mice, potentially through the suppression of glycogen phosphorylase and glucose‐6‐phosphatase activities [15].

According to previous studies, *A. membranaceus* is widely employed to treat CVDs [16]. Studies have reported that As‐IV has a cardioprotective effect on CVDs which works consistently through an autophagic mechanism [17]. The As‐IV induces or inhibits autophagy depending on the disease condition. As‐IV enhances or blocks autophagy to alleviate inflammation and disease severity. But As‐IV also enhances or blocks autophagy to reduce the disease severity that has no relation with inflammation [17].

In this meta‐analysis, we shed light on the protective effects of As‐IV on IHD. A panoply of mechanisms has been recorded recently after the previous meta‐analysis in 2018. The previous meta‐analysis covered myocardial infarct size, LVEF, LVFS, LDH, vascular endothelial growth factor (VEGF), microvessel density, apoptosis rate, and coronary blood flow. Compared to the previous meta‐analysis, we have explored additional parameters, and some of the beneficial outcomes of As‐IV on IHD are reductions in infarct size [18], heart weight/body weight (HW/BW) [19], and left ventricular weight/body weight. As‐IV also demonstrated an increase in Bcl‐2 expression, reduced Bax expression [20], reduction in apoptosis [21], increased LVSP [22], decreased LVEDP [23, 24], increased fractional shortening (FS) [25] and EF [23]. Additionally, As‐IV has decreased LDH release and apoptosis [21], modulated p‐ERK/ERK signaling [26], and reduced CK levels [23], and reduced expression of TLRs, interleukin‐1 (IL‐1), TNF‐α, and NF‐κB. Other parameters like VEGF, p‐Akt/Akt expression, Collagen I and III, and IL‐6 were also been explored. This meta‐analysis has reconfirmed the already analyzed parameters from the previously published meta‐analysis by additional studies and has also explored more number of parameters on apoptosis, inflammation, and cardioprotection, which may possibly uncover the mechanism of action.

## 2 Materials and Methods

### 2.1 Search Strategy and Data Source

A comprehensive literature search was conducted using Medline (via PubMed), Google Scholar, and EMBASE platforms. Moreover, reference lists of eligible studies were checked to identify all preclinical experimental investigations about the effects of As‐IV on IHD. The search combined both Medical Subject Headings (MeSH) terms and keywords; using “Astragaloside IV,” “Ischemic Heart Disease,” “Myocardial ischemia,” “Myocardial reperfusion” as MeSH‐term/keyword, with free text words similar to Thesaurus‐indexing‐procedures to find relevant work. Cross‐references of the identified papers and reviews were also searched to include all available information. ACP search terms “Astragaloside IV,” “ischemic heart disease,” and cardioprotection AND myocardial ischemia were applied while performing literature search in comprehensive electronic databases. We performed the literature search from October 15, 2023 to March 31, 2024.

### 2.2 Inclusion Criteria

Only preclinical research studies were considered based on the total number of records found. In addition to the findings reported in 2018 published meta‐analysis, the current review also emphasizes recent advancements and emerging data published thereafter.

### 2.3 Exclusion Criteria

1. Studies with only in vitro data.
2. Review articles, book chapters, thesis, conference proceedings.
3. Studies with no/incomplete data.
4. Studies involving Astragaloside derivatives, combinations with other drugs/herbs.
5. Studies not in English.
6. Studies with no full‐text papers.

### 2.4 Parameters

The study evaluated various parameters associated with cardiovascular conditions to comprehensively assess the impact of As‐IV treatment. Physical parameters included myocardial infarct size, HW/BW ratio, and left ventricular weight/body weight ratio. Physiological and functional parameters assessed were left ventricular systolic pressure (LVSP), left ventricular end‐diastolic pressure (LVEDP), left ventricular internal dimensions during systole and diastole, ejection fraction (EF), and fractional shortening (FS). Biochemical parameters such as creatinine kinase and lactate dehydrogenase were considered to evaluate myocardial injury. Apoptotic parameters including apoptotic rate, caspase‐3, Bcl‐2, and Bax were analyzed to determine the extent of programmed cell death. Inflammatory parameters were assessed to understand the inflammatory response, including IL‐1, interleukin‐6 (IL‐6), TNF‐α, NF‐κB, TGF‐β, and toll‐like receptor‐4 (TLR4). Parameters such as VEGF and collagen types I and III were examined to evaluate tissue remodeling and angiogenesis. Antioxidant parameters, specifically superoxide dismutase (SOD) activity, were analysed to assess oxidative stress. Additionally, signaling mechanisms involving the p‐Akt/Akt and p‐ERK/ERK pathways were investigated to elucidate the molecular pathways affected by the treatments.

### 2.5 Data Collection and Analysis

Two reviewers have independently scrutinized all the studies based on the inclusion and exclusion criteria. The analysis was performed initially at the title and abstract level and later applied to the entire text as well. Any conflicts between the reviewers were resolved by consulting with a third reviewer. The data extracted from the studies included information about experimental conditions, animals, methods used, interventions, and outcome measurements. Plotdigitizer (https://plotdigitizer.com/) was utilized to extract numerical data from graphs. The high dose was considered in studies where multiple doses of As‐IV were used. When standard deviation (SD) or standard error of the mean (SEM) were unavailable, a default value of 10% of the mean was considered as SD for both As‐IV and control groups, instead of assigning zero.

### 2.6 Assessment of Risk of Bias

The risk of bias assessment tool to evaluate the methodological quality of the chosen studies was the Systematic Review Centre for Laboratory Animal Experimentation (SYRCLE). This tool consists of ten entries related to selection bias, performance bias, detection bias, attrition bias, reporting bias, and other biases. The evaluation of each item was based on the following criteria: low risk of bias, high risk of bias, and moderate risk of bias.

### 2.7 Statistical Analysis

We used Review Manager (RevMan 5.4.1) for the meta‐analysis. A standardized mean difference (SMD) was used for analysing the continuous variables, and we estimated the inverse variance (IV) as an effect measure using the random‐effects model. For dichotomous variables, we employed the Mantel–Haenszel (M–H) statistic with an odds ratio (OR) or risk ratio (RR) as the effect measure, using both random‐effects (RE) and fixed‐effects (FE) models. A RE model was used to calculate the pooled prevalence, with a 95% confidence interval (CI).

The *I* ^2^ statistic was used to evaluate the heterogeneity. This test evaluates the proportion of diversity (heterogeneity) between research findings attributable to variety instead of random sampling error. A value of *I* ^2^ of less than 40% was deemed insignificant. Conversely, moderate to high heterogeneity was defined as an *I* ^2^ value greater than 40%. A sensitivity analysis was conducted to assess the reliability of the results.

## 3 Results

### 3.1 Selection of Articles

**Figure 1:**
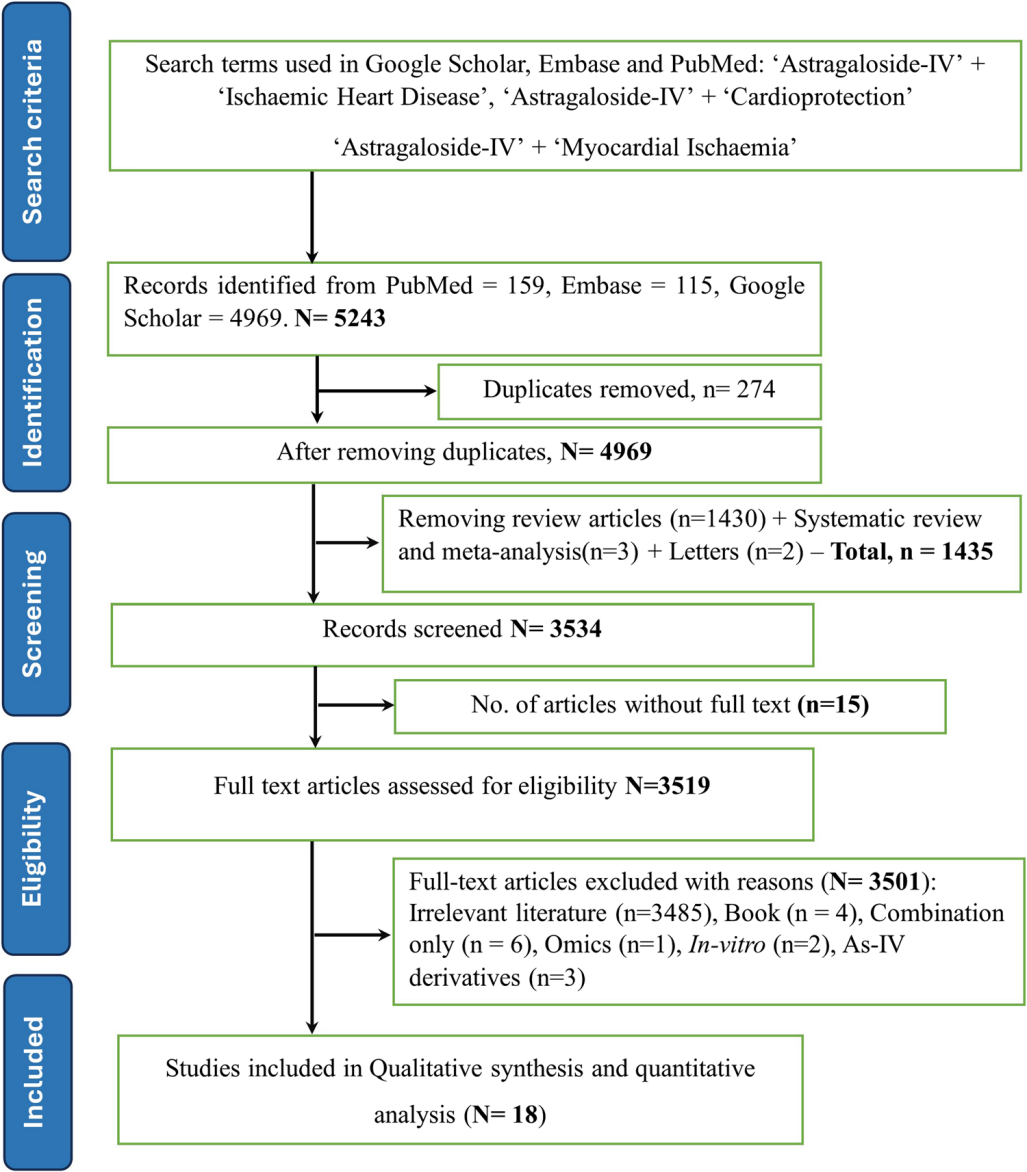
PRISMA flow chart.

A total of 5243 articles were identified using predetermined criteria across various databases, including PubMed/Medline (*n* = 159), Embase (*n* = 115), Google Scholar (*n* = 4969), and other sources (*n* = 0) up to March 31, 2024. Of these, 4969 papers were screened after removing 274 duplicates. Additionally, 1430 review articles (All types of reviews, including any articles where As‐IV was referenced or discussed), 3 systematic reviews and meta‐analyses, 2 letter to the editor articles were excluded. Furthermore, 15 papers were unavailable in full text, and 3501 papers were excluded based on other criteria. Ultimately, 18 preclinical research studies were selected for the systematic review and meta‐analysis. Figure 1 and Table 1 provide an overview of the search strategy and details of the studies included in this review.

**Table 1:** Description of included studies.

References	Animal species (no. of animals/group)	Model employed	Dose, route, and duration of treatment (As‐IV)	Control	Parameters	Title
Zhang et al. [27]	Mongrel dogs (male, *n* = 6/group)	LAD ligation	As‐IV (1.5 mg/kg, i.v.)	DanShen injection (15 mg/kg)	Myocardial infarct size	Astragaloside IV from *Astragalus membranaceus* shows cardioprotection during myocardial ischemia in vivo and in vitro
Zhao et al. [28]	Wistar rats, *n* = 10/group	LAD ligation	As‐IV (1.0 mg/kg, i.v.) for a period of 3 weeks, following a coronary ligation, is recommended to take once a day for 14 days	Equal volumes of normal saline intravenously	Left ventricular internal dimension—systole and diastole, left ventricular systolic pressure, left ventricular end‐diastolic pressure, fractional shortening %, apoptosis rate, Bax expression	Effects of Astragaloside IV on heart failure in rats
Wang et al. [25]	Wistar rats (male, *n* = 9–10/group).	LAD ligation	As‐IV (40 mg/kg, oral) for 4 weeks	Treated with equal volumes of distilled water	Fractional shortening %, ejection fraction	Astragalosides rescue both cardiac function and sarcoplasmic reticulum Ca^2+^ transport in rats with chronic heart failure
Tu et al. [18]	SD rats (male, *n* = 6/group)	LAD ligation for 30 min, then reperfusion for 90 min	As‐IV (10 mg/kg, oral) in saline 90 min before ischemia	Normal saline at 1 mL/kg, oral	Left ventricular end‐diastolic pressure, myocardial infarct size	Astragaloside IV protects the heart from ischemia and reperfusion injury via energy regulation mechanisms
Lu et al. [29]	SD rats (male, *n* = 10/group)	LAD ligation for 30 min, then reperfusion for 2 h	As‐IV (80 mg/kg, oral) suspended in 0.5% sodium carboxymethylcellulose daily for 7 days	0.5% sodium carboxymethylcellulose for 7 days, orally	Myocardial infarct size, apoptosis rate, caspase‐3 expression, Bax expression, TLR4 expression, NF‐κB expression, IL‐1 levels	Astragaloside IV attenuates injury caused by myocardial ischemia/reperfusion in rats via regulation of toll‐like receptor 4/nuclear factor‐κB signaling pathway
Yu et al. [30]	Wistar rats (male, *n* = 8/group)	LADCA ligation	As‐IV (10 mg/kg, i.p.)	Equal volume of normal saline intraperitoneally	Left ventricular mass index, VEGF	Astragalosides promote angiogenesis via vascular endothelial growth factor and basic fibroblast growth factor in a rat model of myocardial infarction
Cheng et al. [31]	SD rats (male, *n* = 15/group)	LAD ligation	As‐IV (50 mg/kg/day, i.v.) for 14 days before model established	Normal saline intravenously for 14 days before model established	NF‐κB expression, TLR4 expression, Bax expression, Bcl‐2 expression, ejection fraction, fractional shortening %, left ventricular internal dimension during systole and diastole, heart weight to body weight ratio, myocardial infarct size	Astragaloside IV enhances cardioprotection of remote ischemic conditioning after acute myocardial infarction in rats
Wang et al. [32]	SD rats (*n* = 6/group)	LADCA ligation	As‐IV (20 mg/kg, i.v.) for 14 days	Equal volume of normal saline intravenously	Left ventricular mass index, LVIDs, LVIDd, FS%, EF%, TGF‐β, Collagen I and III	Tetramethylpyrazine and Astragaloside IV synergistically ameliorate left ventricular remodeling and preserve cardiac function in a rat myocardial infarction model
Yin et al. [21]	SD rats (male, *n* = 8/group)	LAD ligation for 30 min followed by 120 min reperfusion	As‐Ⅳ (80 mg/kg, oral) dissolved in 0.5% sodium carboxymethylcellulose for 7 days prior to inducing ischemia	0.5% sodium carboxymethylcellulose orally	Myocardial infarct size, creatinine kinase, lactate dehydrogenase, apoptosis rate, caspase‐3 expression, Bcl‐2 expression, Bax expression, p‐ERK/ERK expression	Astragaloside IV attenuates myocardial ischemia/reperfusion injury in rats via inhibition of calcium‐sensing receptor‐mediated apoptotic signaling pathways
Jiang et al. [33]	SD rats (male, *n* = 8/group)	Proximal left anterior descending coronary artery	As‐IV (40 mg/kg, oral) for 10 min for 7 days	Normal saline 10 mL/kg, orally	Myocardial infarct size, left ventricular development pressure, fractional shortening %, ejection fraction, creatinine kinase, lactate dehydrogenase, p‐ERK/ERK expression, p‐Akt/Akt pathway	Astragaloside IV attenuates myocardial ischemia–reperfusion injury from oxidative stress by regulating succinate, lysophospholipid metabolism, and ROS scavenging system
Cheng et al. [34]	SD rats (male, *n* = 10/group)	Ligation of LAD	As‐IV (50 mg/kg/day, oral) for 2 weeks	Normal saline orally	p‐Akt/Akt pathway, Bax expression, Bcl‐2 expression, ejection fraction, fractional shortening %, left ventricular internal dimension during systole and diastole, myocardial infarct size	Astragaloside IV exerts angiogenesis and cardioprotection after myocardial infarction via regulating PTEN/PI3K/Akt signaling pathway
Wei et al. [23]	SD rats (*n* = 12/group)	Left anterior descending coronary artery ligation	As‐IV (10 mg/kg, oral) dissolved in sodium carboxymethylcellulose for 7 days	1% sodium carboxy methyl cellulose solution, orally	Myocardial infarct size, heart‐to‐body weight ratio, left ventricular systolic pressure, left ventricular end‐diastolic pressure, fractional shortening %, ejection fraction, creatinine kinase, p‐Akt/Akt pathway	Astragaloside IV alleviates myocardial ischemia–reperfusion injury in rats through regulating PI3K/AKT/GSK‐3β signaling pathways
Huang et al. [20]	C57BL mice (male, *n* = 6/group)	LAD ligation	As‐IV (20 mg/kg, i.p.)	Normal saline, intraperitoneally	Creatinine kinase, myocardial infarct size	A single, acute Astragaloside IV therapy protects cardiomyocyte through attenuating superoxide anion‐mediated accumulation of autophagosomes in myocardial ischemia–reperfusion injury
Zhang et al. [19]	C57BL6 (male, *n* = 8/group)	Hypoxia‐induced hypertrophy model	As‐IV (80 mg/kg, oral) dissolved in sodium carboxymethylcellulose	Sodium carboxymethylcellulose, orally	Heart weight to body weight ratio	Astragaloside IV mitigates hypoxia‐induced cardiac hypertrophy through calpain‐1‐mediated mTOR activation
Sun et al. [26]	C57BL mice (*n* = 6/group)	Coronary artery ligation	As‐IV (50 mg/kg/day, oral) for 2 weeks	Phosphate buffer saline, orally	p‐ERK/ERK expression, IL‐1 expression, ejection fraction, fractional shortening %, left ventricular internal dimension during systole, myocardial infarct size	Astragaloside IV ameliorates myocardial infarction‐induced apoptosis and restores cardiac function
Shi et al. [35]	SD (*n* = 10/group)	Coronary artery ligation	As‐IV (80 mg/kg/day, oral) for 2 weeks	Deionized water, orally	Left ventricular systolic pressure, left ventricular end‐diastolic pressure, TLR4 expression, NF‐κβ expression	Astragaloside IV prevents acute myocardial infarction by inhibiting the TLR4/MyD88/NF‐κB signaling pathway
Luo et al. [24]	SD rats (*n* = 8/group)	Isolated heart anoxia/reoxygenation model	As‐IV (5 mg/kg, i.p.)	Phosphate buffer saline, intraperitoneally	Caspase‐3 expression, lactate dehydrogenase levels, left ventricular development pressure, myocardial infarct size	Nutritional preconditioning induced by astragaloside Ⅳ on isolated hearts and cardiomyocytes against myocardial ischemia injury via improving Bcl‐2‐mediated mitochondrial function
Zhai et al. [36]	C57BL/6 mice (male, *n* = 6/group)	LAD ligation	As‐IV (15 mg/kg, i.p.) for 14 days	Phosphate buffer saline, intraperitoneally	Myocardial infarct size, FS%, EF, CK, LDH, caspase‐3, Bcl‐2, Bax, IL‐1, IL‐6, TNF‐α, SOD activity	The combination of Tanshinone IIA and Astragaloside IV attenuates myocardial ischemia–reperfusion injury by inhibiting the STING pathway

The models used to induce ischemia in vivo were LAD ligation model, hypoxia‐induced hypertrophy model, and isolated heart anoxia/reoxygenated model. LAD ligation involves ligating the left anterior descending coronary artery to reduce the blood flow to the heart followed by abrupt reperfusion which triggers an inflammatory response and free radical generation contributing to apoptosis and cell death. Hypoxia‐induced hypertrophy shifts the aerobic glycolysis to anaerobic glycolysis to increase the glycolysis and as a result lactic acidosis may also occur. This leads to increased stress. Moreover, hypoxia can also activate the MAPK pathway, which can lead to hypertrophy, thus causing a mismatch in the oxygen supply and demand. The anoxia‐reoxygenated model also shifts from aerobic to anaerobic glycolysis, thus depleting the ATP production and further altering the sodium‐potassium ATPase and calcium pump function. This leads to cellular swelling, metabolic acidosis, and ROS accumulation. The rapid reoxygenation further promotes the ROS generation and causes oxidative stress.

### 3.2 Risk of Bias

The risk of bias was performed using the SYRCLE risk of bias assessment tool. The SYRCLE's risk of bias tool consists of ten entries based on six related types of bias. Those six biases are selection bias, performance bias, detection bias, attrition bias, reporting bias, and other biases. The ranking criteria depend on the judgment made by the author. The “yes” judgment indicates that the risk of bias is low, and the “no” judgment suggests a high degree of bias risk. If the report's details are insufficient to assess the appropriate risk of bias, the judgment would be “unclear.” All the studies scored a minimum of 2 points and a maximum of 3 points. The risk of bias in the included studies is shown in Table 2.

**Table 2:** SYRCLE's risk of bias assessment for the included studies.

References	Item 1	Item 2	Item 3	Item 4	Item 5	Item 6	Item 7	Item 8	Item 9	Item 10	Score
Cheng et al. [31]	Yes	Unclear	Unclear	Unclear	Unclear	Unclear	Unclear	No	Yes	Unclear	2
Cheng et al. [34]	No	Unclear	No	Unclear	Unclear	Unclear	Unclear	Yes	Yes	Unclear	2
Huang et al. [20]	No	Unclear	No	Unclear	Unclear	Unclear	Unclear	Yes	Yes	Unclear	2
Jiang et al. [33]	Yes	Unclear	Unclear	Unclear	Unclear	Unclear	Unclear	Yes	Yes	Unclear	3
Lu et al. [29]	Yes	Unclear	Unclear	Unclear	Unclear	Unclear	Unclear	Yes	Yes	Unclear	3
Yu et al. [30]	No	Unclear	No	Unclear	Unclear	Unclear	Unclear	Yes	Yes	Unclear	2
Wang et al. [32]	Yes	Unclear	Unclear	Unclear	Unclear	Unclear	Unclear	Yes	Yes	Unclear	3
Luo et al. [24]	Yes	Unclear	Unclear	Unclear	Unclear	Unclear	Unclear	Yes	Yes	Unclear	3
Shi et al. [35]	Yes	Unclear	Unclear	Unclear	Unclear	Unclear	Unclear	Yes	Yes	Unclear	3
Sun et al. [26]	Unclear	Unclear	Unclear	Unclear	Unclear	Unclear	Unclear	Yes	Yes	Unclear	2
Tu et al. [18]	Unclear	Unclear	Unclear	Unclear	Unclear	Unclear	Unclear	Yes	Yes	Unclear	2
Wang et al. [25]	Yes	Unclear	Unclear	Unclear	Unclear	Unclear	Unclear	Yes	Yes	Unclear	3
Wei et al. [23]	Yes	Unclear	Unclear	Unclear	Unclear	Unclear	Unclear	No	Yes	Unclear	2
Yin et al. [21]	Yes	Unclear	Unclear	Unclear	Unclear	Unclear	Unclear	Yes	Yes	Unclear	3
Zhang et al. [19]	Yes	Unclear	Unclear	Unclear	Unclear	Unclear	Unclear	Yes	Yes	Unclear	3
Zhao et al. [28]	Yes	Unclear	Unclear	Unclear	Unclear	Unclear	Unclear	No	Yes	Unclear	2
Zhang et al. [27]	No	Unclear	No	Unclear	Unclear	Unclear	Unclear	Yes	Yes	Unclear	2
Zhai et al. [36]	Yes	Unclear	Unclear	Unclear	Unclear	Unclear	Unclear	Yes	Yes	Unclear	3

*Note:* Ranking criteria: The “yes” judgment indicates that the risk of bias is low, and the “no” judgment indicates a high degree of bias risk. If the details of the report are insufficient to assess the appropriate risk of bias, the judgment will be “unclear.”

Item 1—Was the allocation sequence adequately generated and applied?

Item 2—Were the groups similar at baseline or were they adjusted for confounders in the analysis?

Item 3—Was the allocation adequately concealed?

Item 4—Were the animals randomly housed during the experiment?

Item 5—Were the caregivers and/or investigators blinded from knowledge which intervention each animal received during the experiment?

Item 6—Were animals selected at random for outcome assessment?

Item 7—Was the outcome assessor blinded?

Item 8—Were incomplete outcome data adequately addressed?

Item 9—Are reports of the study free of selective outcome reporting?

Item 10—Was the study apparently free of other problems that could result in a high risk of bias?

A significant proportion of studies were classified as having an “unclear” risk of bias due to insufficient data reporting, which is a common challenge in preclinical studies, unlike clinical studies where stricter reporting guidelines are followed. While all the studies were free from selective outcome reporting, many lacked sufficient data or did not report key items such as allocation concealment, random housing, and blinded assessment. Furthermore, only a few studies provided complete information on randomization procedures. The “unclear” or “high risk” classifications complicate the assessment of internal validity and may introduce systematic bias, potentially inflating or underestimating the observed treatment effects. Adopting more rigorous and standardized reporting practices would enhance the robustness and reproducibility of future animal studies.

### 3.3 Cardiac Parameters

#### 3.3.1 Myocardial Infarct Size

The myocardial infarct size increases during IHD. In the model chosen, the myocardium is first deprived of blood and oxygen, followed by abrupt reperfusion, which accelerates cardiac cell death and increases the infarct size. In the control, there is an increase in the infarct size. Treatment with As‐IV reduces the myocardial infarct size (IV: −19.44 [−24.12, −14.77] at 95% CI, *p* < 0.00001, *I* ^2^ = 87%). The results are depicted as forest plots in Figure 2A. Yu and colleagues have shown a reduction in myocardial infarct size histologically. However, the numerical data are not available [30].

Figure 2Effect of Astragaloside IV on cardiac parameters (A–K).

**Figure:**
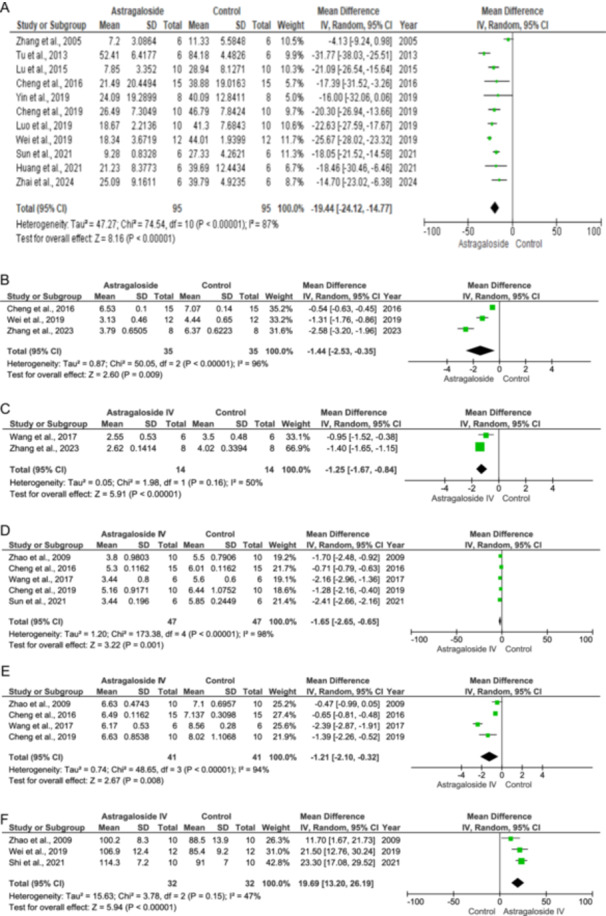

**Figure:**
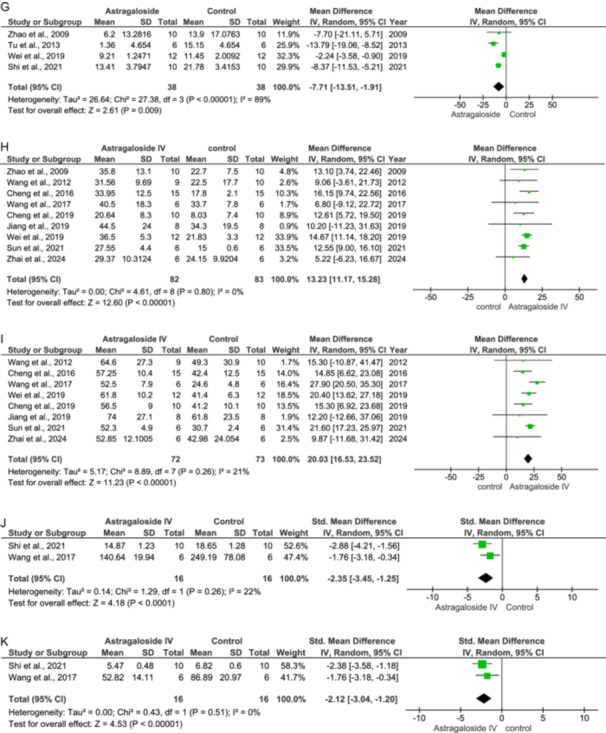

The heterogeneity for this parameter is 87%, indicating significant variability between the studies. This variability may be attributed to differences in sample sizes, doses, dosing schedules, and routes of administration (as shown in Table 1), all of which can influence the statistical power of the analysis. Additionally, baseline infarct sizes in the control groups and infarct sizes after treatment vary significantly across the studies. Notably, Zhang and colleagues used Beagle dogs, while the other studies employed different strains of rats, further contributing to this heterogeneity, as variations in species and strains can impact the biology of the preclinical models [27]. The reported 19.44% reduction in infarct size represents the mean of diverse outcomes, with some studies showing a greater reduction and others a lesser one. Therefore, the true effect of As‐IV may vary across different laboratory settings.

#### 3.3.2 Heart Weight/Body Weight

Ischemic stress leads to the thickening of the heart as a compensatory mechanism to pump blood efficiently, thereby increasing the heart weight–body weight index. Treatment with As‐IV reduces the ratio of HW to BW since the infarct size decreases in the treatment group (IV: −1.44 [−2.53, −0.35] at 95% CI, *p* = 0.009, *I* ^2^ = 96%). Figure 2B illustrates the results through forest plots.

#### 3.3.3 Left Ventricular Weight/Body Weight

The left ventricular size increases during ischemia as a compensatory mechanism against reduced oxygen supply and increased oxygen demand. As‐IV decreases the LVW/BW as shown in Figure 2C (IV: −1.25 [−1.67, −0.84] at 95% CI, *p* < 0.00001, *I* ^2^ = 50%).

#### 3.3.4 Left Ventricular Internal Diameter—Systole and Diastole

The left ventricular internal diameter (LVID) is used to measure the overall size of the left ventricle. In the control group, there is a decrease in the internal diameter during systole and diastole. Upon treatment with As‐IV, a decrease in the LVID during systole (IV: −1.65 [−2.65, −0.65] at 95% CI, *p* = 0.001. *I* ^2^ = 98%) and diastole (IV: −1.21 [−2.10, −0.32] at 95% CI, *p* = 0.008, *I* ^2^ = 94%) can be observed as in Figure 2D,E respectively.

#### 3.3.5 Left Ventricular Systolic Pressure

The LVSP is the pressure generated to pump blood out of the heart to the rest of the body. The control group showed decreased LVSP due to ischemia, while treatment with As‐IV enhanced the LVSP (IV: 19.69 [13.20, 26.19] at 95% CI, *p* < 0.00001, *I* ^2^ = 47%) as shown in Figure 2F.

#### 3.3.6 Left Ventricular End‐Diastolic Pressure

The LVEDP is the pressure generated after the diastolic filling of the ventricles and happens before the systole. From Figure 2G, it can be seen that LVEDP is increased in the control group, while in the treatment group, there is a significant reduction in the LVEDP (IV: −7.71 [−13.51, −1.91] at 95% CI, *p* = 0.009, *I* ^2^ = 89%).

#### 3.3.7 Fractional Shortening %

The FS is a crucial measure of ventricular function in the human heart. FS% reduces during ischemia and the same is reflected in the control group as in Figure 9. However, treatment with As‐IV improves the FS% (IV: 13.23 [11.17, 15.28] at 95% CI, *p* < 0.00001, *I* ^2^ = 0%) as shown in Figure 2H.

#### 3.3.8 Ejection Fraction

The EF is a measure of the amount of blood that is pumped out of the heart with each heart beat. The EF reduced drastically in the control group due to ischemia. However, upon treatment with As‐IV, there is an increase in the EF (IV = 20.03 [16.53, 23.52] at 95% CI, *p* < 0.00001, *I* ^2^ = 21%) as shown in Figure 2I.

#### 3.3.9 Collagen I and III Levels

Ischemic stress enhances the levels of Collagen I and III. Treatment with As‐IV reduces the Collagen I (IV: −2.35 [−3.45, −1.25] at 95% CI, *p* < 0.0001, *I* ^2^ = 22%) and III levels (IV: −2.12 [−3.04, −1.20] at 95% CI, *p* < 0.00001, *I* ^2^ = 0%), as per Figure 2J,K.

### 3.4 Biochemical Parameters

#### 3.4.1 Creatinine Kinase Levels

**Figure 3:**
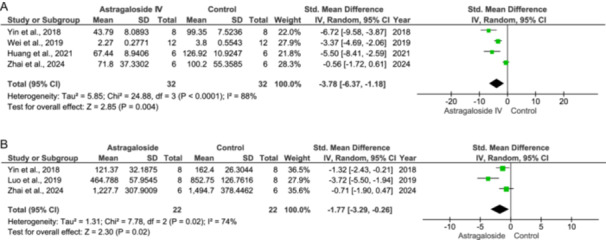
Effect of Astragaloside IV on cardiac biomarkers (A and B).

Whenever there is an insult to the myocardium, the creatinine kinase (CK) levels peak within 6–12 h of the insult. CK is one of the parameters that is used as a biomarker for cardiac diseases. The control group shows increased levels of CK in the serum due to ischemia. However, the treatment group has recorded lower levels of CK in the serum (IV = −3.78 [−6.37, −1.18] at 95% CI, *p* = 0.004, *I* ^2^ = 88%) as per the forest plot generated and depicted in Figure 3A.

#### 3.4.2 Lactate Dehydrogenase Levels

Lactate dehydrogenase is a crucial enzyme that plays a pivotal role in the body's energy‐building process. It is also a biomarker used in the detection of cardiac diseases. The control group shows elevated levels of LDH in the serum due to ischemic conditions. However, treatment with As‐IV reduces the LDH levels significantly (IV: −1.77 [−3.29, −0.26] at 95% CI, *p* = 0.02 *I* ^2^ = 74%), as recorded in the forest plot of Figure 3B.

### 3.5 Apoptotic Parameters

#### 3.5.1 Apoptosis Rate

**Figure 4:**
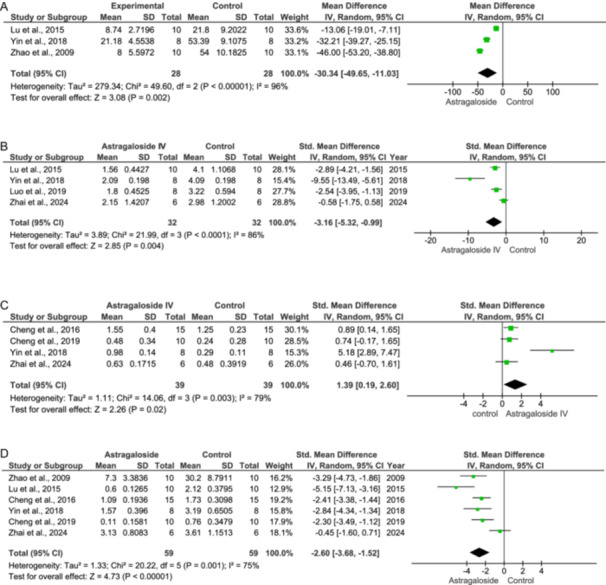
Effect of Astragaloside IV on apoptotic parameters (A–D).

Apoptosis is the process of programmed cell death. During ischemia, due to lack of blood flow to the myocardium, the cells get deprived of oxygen and hypoxia occurs, which further leads to myocardial infarction and cell death. Therefore, the rate of apoptosis is increased during cell death. In the control group, the apoptosis rate is higher, while in the treatment group, it has been observed that As‐IV has managed to control the apoptosis rate (IV: −30.34 [−49.65, −11.03] at 95% CI, *p* = 0.002, *I* ^2^ = 96%.), as shown in Figure 4A.

#### 3.5.2 Caspase‐3 Expression

Caspase‐3 is an apoptosis mediator. It supports the process of programmed cell death. Caspase‐3 is activated by caspase‐8 and caspase‐9. Caspase‐3, once activated, cleaves those proteins like DNA, and repair protein, which, upon cleavage, inhibits the repair process of cells, and also destroys the cell integrity. In the control group, the expression of caspase‐3 levels is higher compared to the treatment group, where As‐IV treatment inhibits the expression of caspase‐3 and reduces the levels of caspase‐3 (IV: −3.16 [−5.32, −0.99] at 95% CI, *p* = 0.004, *I* ^2^ = 86%) as shown in Figure 4B.

#### 3.5.3 Bcl‐2 Expression

The Bcl‐2 is an antiapoptotic factor, and it prevents apoptosis. The control group shows reduced expression while in the treatment group shows a significant rise in the levels of Bcl‐2 (IV: 1.39 [0.19, 2.60] at 95% CI, *p* = 0.02, *I* ^2^ = 79%) as shown in Figure 4C.

#### 3.5.4 Bax Expression

The Bax is a proapoptotic factor, which supports apoptosis. The studies in the control group show that the Bax expression is increased due to ischemia, while in the treatment group, studies have reported a significant decline in the Bax expression (IV: −2.60 [−3.68, −1.52] at 95% CI, *p* < 0.00001, *I* ^2^ = 75%). This has been depicted in Figure 4D.

### 3.6 Anti‐Inflammatory Activity

Abrupt reperfusion often triggers inflammatory responses due to a sudden increase in blood flow and oxidative stress. As‐IV has been reported to have anti‐inflammatory actions according to the meta‐analysis performed.

#### 3.6.1 TLR4 Expression

**Figure 5:**
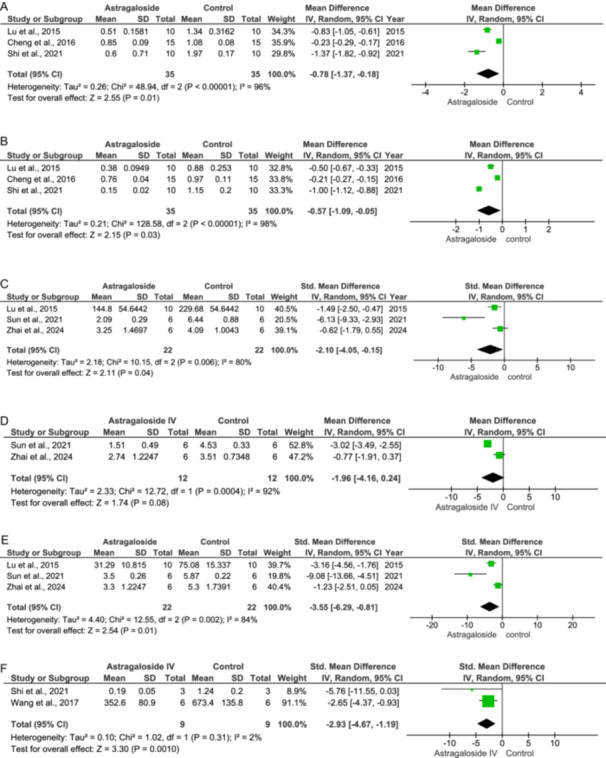
Effect of Astragaloside IV on inflammatory mediators (A–F).

Abrupt reperfusion may trigger an inflammatory response, which aggravates apoptosis and further cardiac cell death (IV: −0.78 [−1.37, −0.18] at 95% CI, *p* = 0.01, *I* ^2^ = 96%), as shown in Figure 5A.

#### 3.6.2 NF‐κB Expression

An increase in the NF‐κB levels has been observed during ischemia. Treatment with As‐IV inhibits the NF‐κB expression (IV: −0.57 [−1.09, −0.05] at 95% CI, *p* = 0.03, *I* ^2^ = 98%), as evidenced in Figure 5B.

#### 3.6.3 IL‐1 Levels

As‐IV reduces the IL‐1 levels significantly compared to the control (IV: −2.10 [−4.05, −0.15] at 95% CI, *p* = 0.04, *I* ^2^ = 80%) as seen in Figure 5C.

#### 3.6.4 IL‐6 Levels

IL‐6 levels increase due to sudden reperfusion. As‐IV decreases IL‐6 levels, however, these actions are not significant (IV: −1.96 [−4.16, 0.24] at 95% CI, *p* = 0.08, *I* ^2^ = 92%) as shown in Figure 5D.

#### 3.6.5 TNF‐α Levels

The levels of TNF‐α increased in the control group, while in the treatment group, the TNF‐α levels decreased (IV: −3.55 [−6.29, −0.81] at 95% CI, *p* = 0.01, *I* ^2^ = 84%), as evident from Figure 5E.

#### 3.6.6 TGF‐β Levels

The TGF‐β levels rise during ischemic stress. Treatment with As‐IV reduced the TGF‐β levels compared to the control (IV: −2.93 [−4.67, −1.19] at 95% CI, *p* = 0.001, *I* ^2^ = 2%) as in Figure 5F.

### 3.7 VEGF Levels

**Figure 6:**
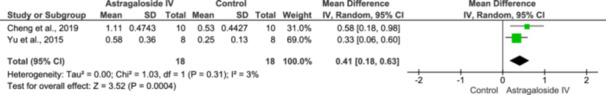
Effect of Astragaloside IV on VEGF.

The VEGF levels decrease during prolonged ischemia. Treatment with As‐IV enhances VEGF levels, supporting angiogenesis (IV: 0.41 [0.18, 0.63] at 95% CI, *p* = 0.0004, *I* ^2^ = 3%). The results are shown in Figure 6.

### 3.8 Antioxidant Activity

**Figure 7:**
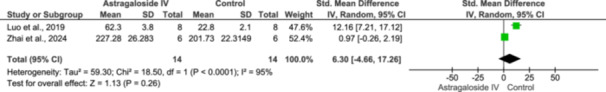
Effect of Astragaloside IV on SOD activity.

The SOD activity drastically decreased during ischemia. However, treatment with As‐IV reverses this and increases the SOD activity, the difference between control and As‐IV group was not statistically significant (IV: 6.30 [−4.66, 17.26] at 95% CI, *p* = 0.26, *I* ^2^ = 95%) according to Figure 7.

In support of As‐IV antioxidant activity, Jiang and colleagues reported an increase in SOD activity in their *ex vivo* experiment, which was not included in this meta‐analysis [33]. This study also observed a decrease in malondialdehyde (MDA) levels, along with increases in catalase and succinate dehydrogenase (SDH) activities, further supporting the antioxidant effects of As‐IV. Additionally, the ratios of Nrf‐2 to GAPDH and Nrf‐2 to lamin B were measured as part of the antioxidative assay, with Nrf‐2/GAPDH showing a decrease and Nrf‐2/lamin B demonstrating an increase. Luo and colleagues reported enhanced ferric reducing antioxidant power (FRAP), SOD activity, glutathione peroxidase (GPx) activity, and catalase activity in an anoxia/regeneration (A/R) model following As‐IV treatment, accompanied by a reduction in MDA levels [24]. Similarly, Zhai and colleagues observed increases in SOD and glutathione (GSH) activity, along with a reduction in MDA levels [36]. These parameters were not included in the meta‐analysis due to the limited number of supporting studies. However, the available evidence from the few studies that reported improvements in these markers suggests that As‐IV exhibits antioxidant effects in the context of IHD in preclinical models.

### 3.9 p‐ERK/ERK Expression

**Figure 8:**
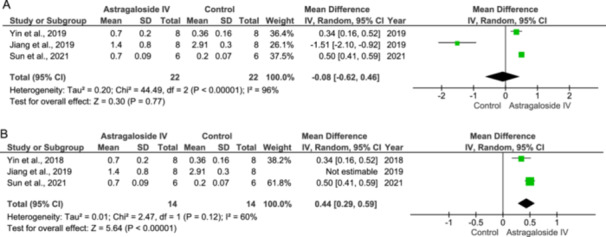
Effect of Astragaloside IV on p‐ERK/ERK expression (A and B).

The As‐IV has modulatory effects on p‐ERK/ERK expression. Jiang and colleagues showed that As‐IV has an inhibitory expression on p‐ERK/ERK expression [33] while the other two studies, Sun et al. [26] and Yin et al. [21], reported that As‐IV increases the expression of p‐ERK/ERK. Hence, the overall values were not statistically significant (IV: −0.08 [−0.62, 0.46] at 95% CI, *p* = 0.77, *I* ^2^ = 96%) compared to the control (Figure 8A).

In the sensitivity analysis, when Jiang et al. [33] were excluded from the analysis, the overall effect of the parameter changed (IV: 0.44 [0.29, 0.59] at 95% CI, *p* < 0.00001, *I* ^2^ = 60%) [33]. This demonstrates the significant influence of Jiang et al. [33] on the overall effect (Figure 8B).

The discrepancies observed in the results may stem from differences in the experimental protocols employed. Specifically, Jiang et al. [33] pretreated animals with As‐IV prior to disease induction, whereas Sun et al. [26] and Yin et al. [21] administered As‐IV as a prolonged or post injury treatment [26]. This variation in treatment timing could potentially account for the discrepancies in the observed outcomes.

### 3.10 p‐Akt/Akt Expression

**Figure 9:**
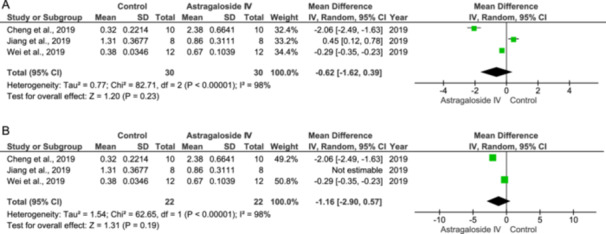
Effect of Astragaloside IV on p‐Akt/Akt expression (A and B).

Jiang et al. [33] reported that As‐IV inhibits p‐Akt/Akt expression, whereas Cheng et al. [34] and Wei et al. [23] reported that As‐IV increases p‐ERK/ERK expression. When combining the results from all three studies, there is no significant effect (IV: −0.62 [−1.62, 0.39], *p* = 0.23, *I* ^2^ = 98%), as shown in Figure 9A. When the study by Jiang et al. [33] is excluded, results are still not statistically significant (IV: −1.16 [−2.90, 0.57], *p* = 0.19, *I* ^2^ = 98%) (Figure 9B).

## 4 Discussion

The As‐IV is a phytochemical obtained from a natural source found to have beneficial effects on the heart. Reports have shown that it can safeguard the heart and prevent cell death through its antiapoptotic actions. Abrupt reperfusion of blood flow to the heart after an ischemic event can trigger a local and systemic inflammatory response that can worsen tissue damage and hinder the recovery of the myocardium [37]. As‐IV suppresses the production of pro‐inflammatory cytokines such as IL‐1, IL‐6, and TNF‐α, thereby exhibiting its anti‐inflammatory properties [29]. This effect is believed to result from the compound's ability to inhibit the TLR4/NF‐κB pathway, which leads to reduced apoptosis of myocardial cells and a further decrease in inflammatory cytokines [29]. Therefore, As‐IV may serve as a potential alternative for preventing heart damage and inflammation associated with IHD.

### 4.1 Cardioprotective Activity

Our meta‐analysis showed that As‐IV is highly effective in reducing the size of myocardial infarcts, probably due to its antiapoptotic properties on the ischemic heart, as reported in earlier studies [21, 28]. Additionally, our findings indicate that As‐IV possesses significant anti‐inflammatory activity, which may help to alleviate ischemia and restore blood flow to the heart [26]. However, more in vivo studies are necessary to confirm this effect in ischemic conditions on the myocardium. Further, studies on cardiomyocytes have demonstrated that As‐IV had improved mitochondrial morphology, contributing to a reduction in infarct size [38].

When ischemia occurs in the heart, the left ventricular myocardial index tends to increase to meet the oxygen demand, leading to left ventricular thickening and, subsequently, declining heart function [39]. Treatment with As‐IV helps restore the balance between oxygen supply and demand in the myocardium, significantly reducing the LVW/BW ratio, which correlates with the HW/BW ratio.

In myocardial ischemia, the blood flow to the heart is compromised, which leads to an increase in the left ventricular internal dimension (LVID) to compensate and accommodate more blood so that cardiac output can be maintained [40]. This adaptive mechanism benefits short‐term outcomes; however, it can lead to long‐term complications such as impaired relaxation and increased cell death [33, 41]. Our current meta‐analysis indicates that As‐IV improves blood flow to the heart which may help to decrease LVID by enhancing cardiac output and reducing the need for increased blood volume.

Further, earlier studies have demonstrated that As‐IV has anti‐apoptotic effects that can prevent accelerated cell death [26]. Also, As‐IV's anti‐inflammatory properties may enhance blood flow to the myocardium and influence the dilation of the left ventricle [42]. However, more in vivo research is needed to fully understand the potential benefits of As‐IV in treating myocardial ischemia.

During myocardial ischemia, the heart muscle fails to generate sufficient pressure to pump blood effectively throughout the body [43]. This inadequacy is primarily due to increased apoptosis and inflammation [19, 44, 45]. Under these conditions, energy production shifts from aerobic to anaerobic glycolysis, leading to lactic acid production and further worsening heart function [46]. As‐IV can help mitigate these effects by reducing apoptosis and improving blood flow, ultimately increasing LVSP. By decreasing apoptosis, As‐IV promotes a shift back to aerobic glycolysis, enhancing ATP production and generating the necessary pressure for blood circulation [47].

In cases of ischemia, LVEDP often rises due to increased filling pressure, potentially due to enhanced LVID and improved heart muscle relaxation [48]. As‐IV has been shown to improve LVID and blood flow, helping to normalize LVEDP and maintain proper heart function [35].

Reduced ATP production during myocardial ischemia damages cardiomyocytes and compromises cell integrity, causing the leakage of creatine kinase, lactic acid dehydrogenase and other cellular contents into the bloodstream [49]. Detecting these markers in the bloodstream plays a key role in predicting the extent of cardiac damage. Treatment with As‐IV helps restore cell integrity by enhancing ATP production and reducing creatine kinase levels and LDH in the blood exhibiting a cardioprotective role of As‐IV.

Myocardial ischemia often causes an abnormal increase in Collagen I and III levels, contributing to ventricular stiffness and fibrosis, which is primarily due to elevated TGF‐β levels [32]. Treatment with As‐IV can help suppress these collagen levels, promoting physiologically relevant growth and repair of the heart.

### 4.2 Antiapoptotic and Anti‐Inflammatory Activity

Recent studies have highlighted the effectiveness of As‐IV in reducing apoptosis. The data presented in Figure 4B clearly show a significant reduction in caspase‐3 expression, a key mediator of apoptosis. Concurrently, the levels of Bcl‐2, an anti‐apoptotic protein, were found to increase (Figure 4C), whereas the levels of Bax, a pro‐apoptotic protein, were reduced (Figure 4D). These findings indicate that As‐IV may offer promising therapeutic potential for conditions characterized by increased apoptosis.

For instance, in animal models with myocardial ischemia/reperfusion (MI/R) injury, a significant upregulation of caspase‐3 and Bax protein expression was observed, along with a decrease in Bcl‐2 expression. However, upon treatment with As‐IV, the expression levels of caspase‐3 and Bax decreased, while Bcl‐2 levels rose, suggesting that As‐IV may have beneficial effects in managing MI/R injury [28, 31, 50, 51, 52].

While the exact mechanism of action is not yet fully elucidated, some studies propose that As‐IV may activate the PI3K/Akt pathway, which is known to play a critical role in cell proliferation and survival [53]. This could potentially lead to an increase in Bcl‐2 expression. Further, in vivo studies are necessary to thoroughly investigate the impact of As‐IV on this pathway.

Additionally, there is evidence suggesting that As‐IV may inhibit the activity of p53, a tumor suppressor protein that regulates Bcl‐2 expression in cancer [54]. Normally, activation of p53 leads to the inhibition of Bcl‐2, whereas its suppression can result in the upregulation of Bcl‐2 [55]. However, more comprehensive in vivo studies are needed to confirm these potential mechanisms.

The exact mechanism through which As‐IV reduces Bax expression is still not completely understood. It is speculated that the activation of the PI3K/Akt pathway, which promotes cell survival and proliferation, may play a role in lowering Bax expression [56]. Moreover, the compromised integrity of cardiomyocytes may cause Bax to be released, allowing it to interact with the mitochondrial membrane and trigger apoptosis [57].

Recent research findings are especially noteworthy, as they show a reduction in the size of the affected myocardial area and an improvement in the overall structure of the heart. These observations suggest that the early antiapoptotic effects of As‐IV significantly contribute to reducing subsequent myocardial necrosis, underscoring its potential therapeutic value in treating myocardial injuries.

In the context of inflammation and its regulation during myocardial ischemia/reperfusion (MI/R) injury, TLR4 has been identified as a key mediator, with its deficiency shown to significantly reduce MI/R‐induced damage, lipid peroxidation, and complement deposition [58]. TLR4 activation triggers the NF‐κB signaling pathway by promoting the degradation of IκBα, allowing NF‐κB to translocate to the nucleus and upregulate pro‐inflammatory cytokines such as TNF‐α, IL‐1, and IL‐6 [59, 60, 61]. This cascade amplifies the inflammatory response and contributes to tissue injury. However, pharmacological inhibition of NF‐κB or stabilization of IκBα has been shown to reduce infarct size, preserve cardiac function, and attenuate inflammation and apoptosis, as indicated by decreased caspase‐3 and Bax expression and increased Bcl‐2 levels [62, 63]. Additionally, MI/R elevates TGF‐β levels, which promote collagen I and III accumulation and myocardial stiffness. Treatment with As‐IV reduces TGF‐β expression, demonstrating its anti‐inflammatory and antifibrotic effects.

### 4.3 Angiogenesis

Angiogenesis is the process where new blood vessels grow from an existing vasculature. In a healthy myocardium, VEGF levels are usually low. However, during myocardial ischemia, VEGF levels increase as part of a negative feedback mechanism. This increase aids in forming new blood vessels under ischemic conditions, supporting the growth and repair of vessels and tissues. Treatment with As‐IV upregulates the VEGF levels and promotes angiogenesis [30]. Previous studies have suggested that Akt and VEGF can activate the PI3K/Akt pathway along with endothelial‐Nitric Oxide Synthase (eNOS), which is involved in injury and repair. Apart from these actions, inflammatory and apoptotic responses are also attenuated. Moreover, the actions that promote angiogenesis have been well documented. Numerous scientific studies have shown that activating the VEGF–Akt–eNOS signaling pathway is closely associated with protective effects on the heart following a heart attack (MI). VEGF is a key stimulant and survival factor for endothelial cells, recognized as one of the most potent promoters of new blood vessel formation. It plays a vital role in both angiogenesis, where new blood vessels form from existing ones, and vasculogenesis (formation of new blood vessels). The VEGF activates the PI3K/Akt signaling pathway, leading to increased eNOS expression. eNOS is activated through phosphorylation at the serine1177 residue, producing nitric oxide, which encourages endothelial cell migration and angiogenesis. These processes are essential for injury response and tissue repair, and they are closely related to the development of collateral circulation in coronary arteries [64]. Treatment with As‐IV boosts VEGF levels, enhancing angiogenesis and supporting repair and growth, thereby significantly mitigating myocardial ischemia.

### 4.4 Antioxidant Activity

As previously mentioned, sudden reperfusion during ischemia triggers oxidative stress and the generation of reactive oxygen species (ROS). The excessive production of ROS is believed to play a crucial role in the pathological processes linked to ischemia, reperfusion, and the postreperfusion phase of I/R injury. The ROS have been found to have both harmful and beneficial effects in I/R, contributing to tissue damage that can lead to cognitive impairments in stroke and the enlargement of infarct size due to their presence during ischemia and reperfusion. Simultaneously, they act as signaling molecules that promote fibrosis, angiogenesis, and vascular remodeling during the repair phase [65]. According to our findings, although As‐IV increases the SOD activity, it is not significant enough to conclude that As‐IV exerts antioxidant effects during an ischemic event, and hence more studies are required in this area to confirm this activity.

### 4.5 ERK1/2 Modulatory Activity

The ERK1/2 cascade is one of the most critical pathways in the MAPK family. This is because it regulates cellular activities everywhere, from entry to differentiation, transcription, and survival. Its importance lies in protecting myocardial cells against damage from myocardial infarction and reperfusion, making it a key factor in maintaining heart health [66]. Moreover, the ERK1/2 cascade family also participates in ischemic preconditioning and delayed pharmacological preconditioning phenomena, which provide significant protection for the heart against ischemic injury [67]. Numerous studies show that As‐IV can impact the expression and production of p‐ERK/ERK. Some studies found that As‐IV inhibits p‐ERK/ERK expression, while others observed a pronounced increase in those expression levels. This increment may be due to the inhibition of the calcium‐sensitive receptor pathway coupled with the activation of the MAP kinase pathway in turn [21]. Overall, the ERK1/2 cascade pathway and its regulation by As‐IV are pivotal for heart health, providing the organ with crucial protection against the dangers of ischemic attack. Some changes in parameter scores related to the heart have occurred in studies where various dose levels of As‐IV were used. Improvements in cardioselective parameters such as LVID, LVSP, LVEDP, EF, and FS% have been noted in meta‐analysis. These improvements are achievable by As‐IV's anti‐inflammatory and antiapoptotic effects. However, more work is needed to confirm its effects on the left ventricular development pressure, p‐ERK/ERK expression, and p‐Akt/Akt expression.

In the present study, the results for p‐ERK/ERK and p‐Akt/Akt expression were not statistically significant, preventing us from drawing a conclusive statement regarding the regulatory role of As‐IV. The variability in results can be attributed to differences in experimental procedures, dosing regimens, and the animal models used. The sensitivity analysis demonstrated how significantly the results shift when excluding the study by Jiang et al. [33], highlighting that the findings are highly dependent on the specific methodologies employed in individual studies. Further research is needed, particularly focusing on both pretreatment and postdisease treatment with As‐IV, to confirm the exact mechanisms by which As‐IV influences these parameters.

**Figure 10:**
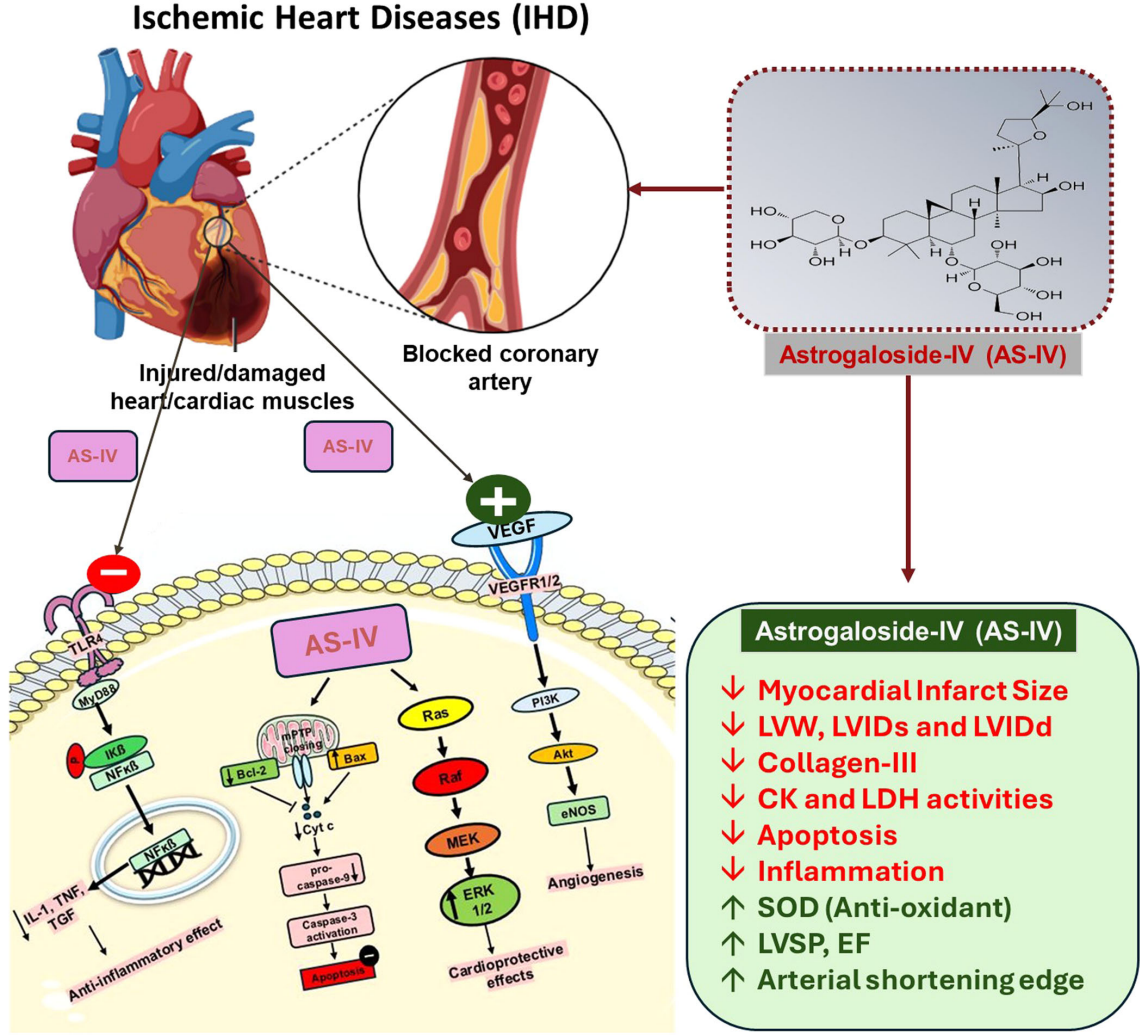
This figure illustrates various mechanisms underlying the cardioprotective activity of Astragaloside IV in ischemic heart disease models.

Figure 10 illustrates the various mechanisms underlying the cardioprotective activity of As‐IV in IHD models.

## 5 Conclusion

This study demonstrated the beneficial effects of As‐IV in multiple models and uncovered its hidden prospects as a cardioprotective agent for IHD. The results of the meta‐analysis show that As‐IV contributes to its cardioprotective properties by managing cell death, inhibiting inflammation biological processes, and promoting angiogenesis in addition to regulating key signaling pathways. As‐IV almost completely abrogates myocardial infarct size and preserves the fundamental heart functions such as LVSP, EF, and FS. This leads not only to a restoration in the oxygen demand and supply but also improves mitochondrial morphology and enhances coronary flow reserve (blood flux) for healthy heart tissue.

The cardioprotection by As‐IV was due to its antiapoptotic effects, indicated by the lowered activity of caspase‐3 and the expression level of Bax and elevation in protein levels of Bcl‐2, which helps it play an important role for preventing cell death during myocardial ischemia. Similarly, its anti‐inflammatory effect via the TLR4/NF‐κB pathway and pro‐inflammatory cytokines inhibition further suggests that As‐IV is beneficial to protect the myocardium from inflammation injury.

Moreover, As‐IV can promote angiogenesis by increasing VEGF expression and improving blood flow to the heart muscle. This is because it is involved in the ERK1/2 cascade, which is significantly associated with cardioprotection against ischemic injury.

Although the results are promising, The LVEDP levels and expression of p‐ERK/ERK, as well as p‐Akt/Akt, still need to be explored for As‐IV pretreatment‐related cardioprotection. In addition, more in vivo studies are needed to verify the possible therapeutic benefits of As‐IV for clinical applications. In conclusion, As‐IV may effectively act as a multitarget alternative medicine to protect the heart from ischemic damage. As‐IV is a key candidate for treating and preventing IHD because it has been demonstrated to reduce apoptosis, anti‐inflammatory effects, and antioxidative stress capacity while showing both proangiogenesis properties supportive of cellular survival pathways. This makes As‐IV a strong candidate for treating and managing IHD.

## Authors' Perspectives

Although As‐IV shows positive effects in animal models of IHD, such as the left anterior descending coronary artery ligation and hypoxia‐induced hypertrophy, there are still some lacunas in the preclinical studies.

First, there are a few parameters that have been assessed qualitatively rather than quantitatively (e.g., myocardial infarct size by Yu et al. [30]), making it difficult to perform any detailed meta‐analysis. In addition, existing research shows considerable heterogeneity, underscoring the requirement for well‐controlled investigations to formalize and enhance our understanding of As‐IV's cardioprotective effects.

The current preclinical evidence is not sufficient to support a comprehensive meta‐analysis of several key parameters. Although available SOD data offer some insight into the antioxidant mechanisms of As‐IV, further studies are necessary to clarify its broader antioxidant pathways in cardioprotection. Existing histopathological assessments are predominantly qualitative, emphasizing the need for standardized quantitative evaluations. Additionally, data on heart rate modulation, autophagosome accumulation, and the involvement of calcium‐sensitive receptors in the cardioprotective effects of As‐IV remain limited, warranting more focused preclinical investigations.

Finally, though abundant preclinical evidence and some in vitro studies are readily available, there needs to be more randomized controlled trials (RCTs) among human populations to verify the results identified for As‐IV on preventing or treating IHD. For As‐IV, therefore, it is mandatory to perform RCTs in the human population before definitive conclusions can be derived regarding its possible future clinical applications.

In conclusion, clinical studies with rigorous methodologies will fill these gaps, supporting a greater understanding of As‐IV and exploring its therapeutic value in IHDs.

## Author Contributions

Alosh Greeny, Gollapalle Lakshminarayanashastry Viswanatha, Rekha Raghuveer Shenoy, Shylaja Hanumanthappa, Dinesh Kumar Chellappan, Jagnoor Singh Sandhu, Saumya Khanna, and Nandakumar Krishnadas contributed significantly to the conceptualization, methodology, investigation, formal analysis, validation, and writing of the original draft.

## Ethics Statement

Ethics approval is not applicable, as this manuscript does not involve any experiments involving either animals or human trials.

## Consent

All the authors have read and approved the manuscript for publication.

## Conflicts of Interest

The authors declare no conflicts of interest.

## Data Availability

The software RevMan 5.4 was freeware.
